# The Potential for a Blood Test for Scabies

**DOI:** 10.1371/journal.pntd.0004188

**Published:** 2015-10-22

**Authors:** Larry G. Arlian, Hermann Feldmeier, Marjorie S. Morgan

**Affiliations:** 1 Department of Biological Sciences, Wright State University, Dayton, Ohio, United States of America; 2 Institute of Microbiology and Hygiene, Charité University Medicine, Berlin, Germany; University of California, San Diego School of Medicine, UNITED STATES

## Abstract

**Background:**

Scabies afflicts millions of people worldwide, but it is very difficult to diagnose by the usual skin scrape test, and a presumptive diagnosis is often made based on clinical signs such as rash and intense itch. A sensitive and specific blood test to detect scabies would allow a physician to quickly make a correct diagnosis.

**Objective:**

Our objective was to profile the mite-specific antibodies present in the sera of patients with ordinary scabies.

**Methods:**

Sera of 91 patients were screened for Ig, IgD, IgE, IgG and IgM antibodies to *S*. *scabiei*, as well as to the house dust mites *Dermatophagoides farinae*, *D*. *pteronyssinus* and *Euroglyphus maynei*.

**Results:**

45%, 27% and 2.2% of the patients had measurable amounts of mixed Ig, IgG and IgE that recognized scabies mite antigens. However, 73.6% of the scabies patients had serum IgM that recognized scabies proteins, and all except two of them also had IgM that recognized all of the three species of dust mites. No patient had serum antibody exclusively reactive to scabies mite antigens.

**Conclusions:**

Co-sensitization or cross-reactivity between antigens from scabies and house dust mites confounds developing a blood test for scabies.

## Author Summary

Scabies, caused by the mite *S*. *scabiei* that burrows in the skin of humans, is a contagious skin disease that affects millions of people worldwide. It is a significant public health burden in economically disadvantaged populations, and outbreaks are common in nursing homes, daycare facilities, schools and workplaces in developed countries. It causes significant morbidity, and in chronic cases, associated bacterial infections can lead to renal and cardiac diseases. Scabies is very difficult to diagnose by the usual skin scrape test, and a presumptive diagnosis is often made based on clinical signs such as rash and itch that can mimic other skin disease. A sensitive and specific blood test to detect scabies-specific antibodies would allow a physician to quickly make a correct diagnosis. Our manuscript reports the antibody isotype profiles of the sera of two groups of patients with ordinary scabies (17 from the US and 74 from Brazil) and concludes that such a blood test should be based on circulating IgM type antibodies that do not also recognize antigens of the related and ubiquitous house dust mites. Both are important considerations for research for developing a blood test for the diagnosis of scabies.

## Introduction

Scabies, caused by the mite, *Sarcoptes scabiei*, is a worldwide-occurring parasitic skin disease [1, 2]. It was recently added to the list of neglected tropical diseases by the World Health Organization [3]. Estimates of the prevalence of scabies range from a small percent of the population in developed countries to high prevalence in some resource-limited communities in countries of the global south where the disease may affect up to 50% of women and children [4–13]. In addition, outbreaks are reported in nursing homes and daycare facilities (among both workers and clients), as well as in kindergartens, hostels, schools, among colleges students, and in work environments where there is much physical contact between individuals [14–18].

Ordinary—in contrast to crusted—scabies is very difficult to diagnose. Parasitological techniques are rather insensitive and clinically scabies can mimic other skin diseases such as eczema, psoriasis, atopic dermatitis, diaper rash, poison ivy dermatitis and skin reactions to irritating agents such as soaps/detergents, metals, and lotions. A presumptive diagnosis of scabies requires confirmation by recovering mites, mite fecal pellets, and eggs from the corneal layer of the epidermis. In practice though, patients are often diagnosed with scabies based on clinical characteristics such as a rash and intense itch. Since none of the symptoms and signs are pathognomonic, this approach frequently results in a false-positive diagnosis, which in turn exposes the patient to a potentially hazardous treatment. In contrast, crusted scabies (also known as “Norwegian scabies”) is easily recognizable by the hyperkeratosis that manifests with a scaly and thickened (i.e., crusted) strateum corneum and accompanying large mite burden as compared to the low mite burden and rash of ordinary scabies.

A sensitive and specific blood test to detect scabies-specific circulating antibodies would allow a physician to quickly make a correct diagnosis. One of the problems with developing such a test is that many of the antigens from scabies mites cross-react with antigens from the common allergy-causing house dust mites, *Dermatophagoides farinae*, *D*. *pteronyssinus* and *Euroglyphus maynei* that occur worldwide [19–22]. We report here the antibody isotype profiles of the sera of two groups of patients with ordinary scabies (from Brazil and the United States) that recognize scabies and house dust mite antigens and illustrate the confounding problem of cross-reactivity and cross-sensitization.

## Methods

### Ethics statement

Serum from the US patients (17 subjects + positive reference) was collected under Human Subjects Protocol (HSP) #0205 as approved by the Wright State University Institutional Review Board (IRB). Negative control sera were previously provided to us without personal identifiers under protocol SC #2714 approved as EXEMPT under CFR 46.101(b)(4) by the Wright State University IRB.

Serum from the Brazilian patients was provided to Wright State researchers without personal identifiers. The research was approved as EXEMPT under CFR 46.101(b)(4) and was approved under protocol SC #4334 by the Wright State University IRB. The original study was approved by Universidade Federal do Céara—Comité de Ética em Pesquisa, protocol 358/08. The text of the consent form was read out loud in the presence of other household members (for both children and adults) and the collection procedures and subsequent laboratory techniques were explained in plain language. All adult subjects and guardians of minor children then signed the consent forms that had been read to them.

### Human sera

Sera were obtained following consent from two groups of patients with ordinary scabies at the time of initial diagnosis. Sera were collected from patients at a dermatologist’s office in Cincinnati, OH, USA. This group was composed of 17 subjects (6 females + 11 males, 18–72 yrs of age) who had ordinary scabies confirmed by the recovery of live mites by skin scraping at the time of diagnosis and who reported having had symptoms for 0–13 months prior to diagnosis. Sera were also collected from 74 scabies patients (44 females + 30 males, 5–73 yrs of age) in a resource-poor community in Fortaleza, Northeast Brazil. These patients were identified by active case detection. The clinical diagnosis was confirmed by dermoscopy and skin scraping and 86.3% of the patients had 3 or more topographic areas affected. In 42.1% the duration of the infestation was < 3 weeks.

As a positive reference, serum previously collected from an ordinary scabies patient was used. This 72 yr old male who presented at a dermatologist’s office in Dayton, OH reported having had scabies symptoms for > 4 yrs. His serum had previously been demonstrated to contain high levels of circulating antibody to scabies [23] and radioallergosorbent testing (RAST; conducted by Clinical Immunology and Allergy, Liberty, MO) showed total IgE > 1000 U/mL and modified RAST class 2 scores (scores range from 0 to 6) for specific IgE to both *D*. *farinae* and *D*. *pteronyssinus*. A pool of negative control sera was prepared by mixing equal volumes of serum from two individuals that had no known history of scabies and that had previously been demonstrated to have low levels of circulating antibody to extracts of any of 9 astigmatid mite species [24, 25].

### Mite extracts

Aqueous extracts were prepared from *Sarcoptes scabiei* var. *canis* and from the house dust mites *Dermatophagoides farinae*, *D*. *pteronyssinus*, and *Euroglyphus maynei* according to our standard protocol. Briefly, scabies mites were collected by aspiration onto a 38 μm stainless steel mesh (Small parts, Inc., Miami Lakes, FL) after they had migrated from crusts. To remove host material, live mites were washed by drawing sequential 4 mL aliquots of PBST (Dulbecco’s Phosphate Buffered saline with 0.05% Tween 20), endotoxin-free water and 70% ethanol through the mesh. Mites were killed by freezing at -80°C where they were stored until used.

Dust mites were collected by aspiration as they migrated from cultures, killed by freezing, lyophilized and stored at -20°C until used. Mites of each species were suspended in endotoxin-free water (at 1:10 W:V for dust mites and 1:20 W:V for scabies mites) for overnight extraction at 4°C. The next day, samples were ground 10 strokes on ice using a TenBroeck homogenizer, the insoluble material was removed by centrifugation for 10 min at 14 k x *g* and the supernatants were sterile filtered into sterile vials. To extract as much protein as possible, the scabies mite pellet was subjected to a second extraction/homogenization/centrifugation and the two supernatants were combined. Protein content in each extract was determined by the method of Bradford [26] using bovine serum albumin (BSA) as the standard.

### Enzyme-linked immunosorbent assays (ELISAs)

Microtiter plates for each mite extract were coated with 0.5 μg protein/well in 50 mM carbonate/bicarbonate buffer, pH 9.6, and allowed to dry. Plates were made in two batches (one for each serum cohort) and were stored in a desiccator at room temperature until used. Before use and between all steps, plates were washed with PBST. Plates were blocked with 1% BSA in PBST and loaded with 100 μL of serum diluted 1/10 (IgD and IgE) or 1/1000 (Ig, IgG and IgM). All samples were loaded in duplicate wells and each plate had a parallel set of positive a4nd negative control sera.

Serum antibody binding was detected using biotinylated antibodies specific for human mixed Ig [IgM+IgG+IgA, H+L], IgD [δ chain specific], IgE [specific for Fc portion of the heavy chain], IgG [γ chain specific], or IgM [μ chain specific] (all diluted 1/5000) followed by streptavidin-peroxidase (1/5000). All were purchased from Southern Biotech (Birmingham, AL). Plates were developed with 100 μL of 1 mM ABTS in 70 mM citrate phosphate buffer, pH 4.0, with 0.03% hydrogen peroxide. Development was stopped by the addition of 50 μL of 0.2% sodium azide and plates were read immediately.

To account for plate-to-plate variability, all data were normalized at the conclusion of the study. For each antibody class and antigen, the 405 nm absorbances of all the wells for the positive control serum were averaged. These values were then used, along with the controls for each individual plate, to calculate normalized ELISA absorbance values for each serum/antibody class/antigen.

## Results

For this study, the negative control serum used was a pool of individuals that had previously been determined to lack circulating antibodies that recognized antigens present in extracts of scabies, house dust and stored product mites [24, 25]. A positive titer to any mite extract was defined as an ELISA absorbance of at least 2 times that of the negative controls for mixed Ig and IgG or exceeding 0.100 for IgM or 0.050 for IgE. The positive reference serum was from a single patient that had scabies for more than 4 years and that had previously been determined to have high levels of antibodies to scabies and *Dermatophagoides* mites.

**Fig 1:**
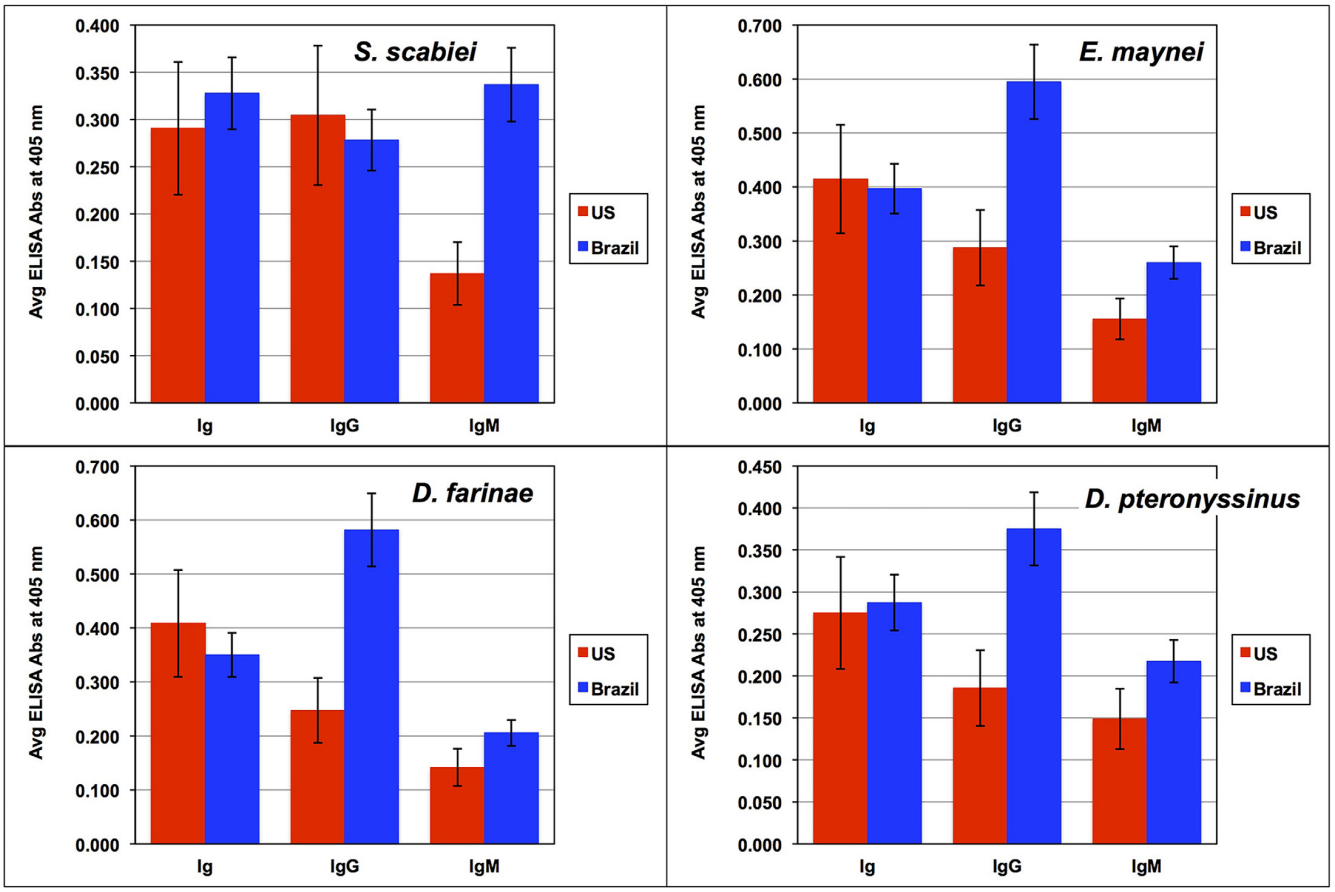
Mixed Ig, IgG and IgM serum antibody binding to ELISA plates coated with scabies or house dust mite antigens. Data are presented as the mean ELISA absorbance ± standard error of the mean for sera collected from ordinary scabies patients in the US (n = 17) and Brazil (n = 74).

ELISA analysis of the serum from 91 scabies patients showed that 45.1% had circulating antibody (Ig) that bound to scabies antigens (Table 1). This included 6 of the 17 patients from the US (35%) and 35 of the 74 patients from Brazil (47%). Of all the 91 patients, 27.5% and 73.6% had measurable levels of serum IgG and IgM antibody, respectively, that bound to scabies proteins (Table 1). Serum IgM levels to scabies were higher for the Brazilian patients than for the US subjects while their Ig and IgG levels were similar (Fig 1). Notably, the average IgM binding to scabies antigens by the Brazilian patients resulted in ELISA absorbances that were more than twice those exhibited by US patients. Only 2 of the scabies patients (2.2%), both Brazilians, had circulating IgE antibodies that bound to scabies proteins and both of these gave very low absorbance readings (0.070 and 0.073). No patient had measurable levels of serum IgD directed at the antigens of any mite.

**Table 1:** Number and percentage of the 91 ordinary scabies patients with serum antibody that recognized scabies and house dust mite antigens by ELISA.

Antibody Class	Serum Dilution		Target antigen on the ELISA plate			
			*S*. *scabiei*	*D*. *farinae*	*D*. *pteronyssinus*	*E*. *maynei*
**Ig**	1/1000	# (+)	41	80	81	84
		% (+)	45.1%	87.9%	89.0%	92.3%
**IgG**	1/1000	# (+)	25	77	83	79
		% (+)	27.5%	84.6%	91.2%	86.8%
**IgM**	1/1000	# (+)	67	69	76	77
		% (+)	73.6%	75.8%	83.5%	84.6%
**IgE**	1/10	# (+)	2	3	2	3
		% (+)	2.2%	3.3%	2.2%	3.3%

A positive titer to any mite extract was defined as an ELISA absorbance of at least 2 times that of the negative controls for mixed Ig and IgG or exceeding 0.100 for IgM or 0.050 for IgE.

All except two of the 67 patients that had serum IgM antibodies to scabies antigens also had IgM that recognized all of the three species of dust mites, *D*. *farinae*, *D*. *pteronyssinus*, and *E*. *maynei*. Of the 25 scabies patients with IgG to scabies, 24 also had IgG to all three dust mite species. No patient had serum antibodies exclusively reactive to scabies mite antigens.

The Brazilian patients had substantially higher levels of serum IgM recognizing antigens of all four mite species than did the US cohort (Fig 1). Although IgG binding to scabies proteins was the same for the two groups, IgG binding to each of the dust mite extracts was higher for the Brazilian cohort than for the US group. No difference between the groups was observed for Ig binding to any of the mite extracts.

### US patients

Among the 17 US patients, 10 had IgM, 6 had IgG and 6 had Ig that recognized scabies mite antigens. Five patients with elevated IgM antibodies to scabies antigens did not have elevated IgG or mixed Ig to scabies. Conversely, 3 patients had elevated IgG antibodies to scabies but not elevated IgM and 3 patients did not show any antibody binding to scabies mite proteins. No US patient had detectable levels of IgE to scabies mites.

While not all of the US patients had circulating antibody that bound to scabies antigens, all did have elevated levels of at least one antibody class that recognized antigens of the house dust mites *D*. *farinae*, *D*. *pteronyssinus*, and *E*. *maynei*. All 6 patients with Ig to *S*. *scabiei* also had Ig to *D*. *farinae*, *D*. *pteronyssinus*, and *E*. *maynei*. There was also no IgE binding detected to any of the house dust mite extracts.

### Brazil patients

Among the 74 Brazilian patients, 57 had IgM and 19 had IgG that recognized scabies antigens while 35 patients had elevated mixed Ig to scabies. Of the 57 patients that had elevated IgM to scabies, 23 did not have elevated IgG or mixed Ig to scabies antigens. Two of the Brazilian patients had detectable levels of IgE to scabies antigens. Eight of the scabies patients had no detectable circulating antibody binding to scabies antigens.

With the exception of one patient that showed no antibody binding to *D*. *pteronyssinus*, all Brazilian patients had Ig, IgG and/or IgM that bound to antigens of all three house dust mite species. Of the 57 patients that had elevated IgM to scabies all except one also had elevated IgM to *D*. *farinae*, *D*. *pteronyssinus* and *E*. *maynei*. Likewise, of the 19 patients that had elevated IgG to scabies all also had elevated IgG to all three dust mites. Of the 35 patients with mixed Ig that recognized scabies mite antigen, all but two also had Ig that recognized antigen of all three species of dust mites.

## Discussion

Although scabies mites burrow and reside in the stratum corneum of the epidermis, historical studies along with more recent studies clearly indicate that antigens from scabies mites induce a humoral response in the host. Thus, theoretically it may be possible to develop a blood test for scabies based on circulating antibodies that recognize scabies-specific antigens that do not cross-react with house dust mites. We provide a brief history of studies profiling information that leads to the conclusion that scabies can be diagnosed with a simple blood test but that the antigens will need to be carefully selected.

Some early studies reported highly diverging results concerning total serum IgG, IgA, and IgE, and complement C3 and C4 levels in scabies patients compared to control subjects without scabies [27–31]. None of these studies directly investigated whether or not the altered serum immunoglobulin isotype levels were the result of scabies infestation and were specific to scabies mite antigens. Hence, these studies do not allow any conclusion.

Several studies indirectly associated serum antibody concentrations to scabies infestation. These studies showing a relationship between changes in antibody isotype levels during scabies infestation compared to those following successful treatment suggested a cause-and-effect association with scabies infestation although the presence of specific antibodies to scabies antigens were not determined [28, 32, 33]. However, it was not excluded that the scabies patients were concomitantly infected with helminths and that the presence of helminths caused the immunological alterations.

More recent studies using *S*. *scabiei* mite extract or recombinant scabies molecules prepared from mites collected from different host species clearly showed that animal and human hosts build antibodies to specific *S*. *scabiei* molecules [21, 23, 34–42]. Two studies showed that human patients with crusted scabies had elevated total scabies-specific IgE compared to those with ordinary scabies [35, 43]. Walton et al. [43] found that subjects with both ordinary and crusted scabies had elevated antibody levels specific for several recombinant scabies antigens compared to naïve control subjects never exposed to scabies. Additionally, an immunoassay employing a recombinant scabies protein, rSar s 14.3, that corresponds to residues 1263–1655 of the dust mite allergen Der p 14, showed significantly higher IgE binding by plasma from crusted scabies patients than from ordinary scabies patients [44]. The IgE binding by the ordinary scabies patients was also significantly higher than that of controls and no cross-reactivity with the dust mite homolog was observed.

Obtaining sufficient amounts of human scabies mites for research purposes is very difficult. However, use of mites collected from dogs, pigs, and foxes as a source of material for research to develop a diagnostic test and vaccine for human scabies offers a promising alternative. It is not clear if *S*. *scabiei* mites from such hosts are the same species or only subtle genetic variants because *S*. *scabiei* mites from most different host species are morphologically indistinguishable. The most recent molecular study indicated that mites collected from humans could be distinguished from those collected from animals based on sequencing of the 317-bp mtDNA *cox*1 gene but not when several other molecular markers were used [45]. This analysis also suggested that all the mites collected from various animal hosts were monospecific while the mites isolated from humans from different geographical locations clustered into four separate branches representing four different species [45].

More importantly, several studies have shown that there is significant antigenic cross-reactivity between different strains of scabies mites from different hosts but there are also strain-specific antigens [23, 46]. For example, *S*. *scabiei* var. *suis* from pigs, var. *canis* from dogs and var. *hominis* from humans share cross-reacting antigens. Likewise, humans with scabies have circulating antibodies that recognize antigens from *S*. *scabiei* var. *vulpis* from foxes, var. *canis*, and var. *suis* [23, 46]. Haas et al. [46] found that 48% of scabies patients had IgG that recognized antigens from fox mites and patients with greater severity and duration of scabies had significantly higher IgG titers. In addition, Western blotting showed that a 72-year-old male with chronic scabies had IgG that recognized more than 10 antigens from *S*. *scabiei* var. *canis* [23]. Also, serum from 6 patients with crusted scabies had IgE to 11–21 and IgG to 1–7 antigens from *S*. *scabiei* var. *canis* but patients with ordinary scabies had serum IgG and IgE that recognized many fewer antigens [35]. And a crude whole body extract of *S*. *scabiei* var. *vulpes* obtained from red foxes contained antigens recognized by antibodies in serum from scabies infested pigs [47] and chamois [48]. An ELISA using the recombinant Ssλ20ΔB3 antigen from *S*. *scabiei* var. *hominis* detects serum antibody in Iberian red deer, Southern chamois, pigs, and rabbits infected with sarcoptic mange [49–53]. Likewise, several recombinant proteins generated from *S*. *scabiei* var. *suis* were recognized by antibodies in sera of human patients infected with *S*. *scabiei* var. *hominis* [54]. Therefore, using mites collected from various host species provides a means of identifying specific molecules and antibody isotypes that may be useful in developing a diagnostic test for scabies and overcomes the problem of a limited supply of var. *hominis* mites.

The present study using a canine strain of scabies to prepare antigen for use in ELISA, found that only 45%, 27% and 2.2% of 91 patients with ordinary scabies had measurable amounts of mixed Ig, IgG and IgE that recognized scabies mite antigens, respectively. No patient had IgD that recognized scabies mite antigen. However, 73.6% of the scabies patients had serum IgM that recognized *S*. *scabiei* antigens with substantially higher levels observed from the Brazilian patients compared to those from the US. This suggests that the Brazilian patients were diagnosed rather early in the infestation since they had not switched to IgG production that follows an initial IgM response. Conversely, the observation that more patients had IgG to the house dust mites suggests a more chronic exposure to these mites. Based on these results, it appears that a diagnostic test should be based on detecting serum IgM to scabies antigens. Such a test would be beneficial because IgM is the first antibody class produced, before class switching to IgG occurs, so it may allow for earlier diagnosis of scabies. The differences between the responses of the two groups of patients may reflect differences in the strains/species of scabies mites infesting these geographically-distant patients as suggested by the Zhao et al. study [45].

This study again elucidates the problem of co-sensitization or cross-reactivity between antigens from house dust mites that confounds developing a blood test for scabies. House dust mites are the sources of > 25 antigenic proteins [55]. Many of the antigens from house dust mites cross-react with those from scabies mites [19–22]. Also a significant percentage of people are sensitized to the ubiquitous house dust mites, *D*. *farinae*, *D*. *pteronyssinus* and *E*. *maynei*. Every scabies patient of this study except one had circulating Ig, IgG, and/or IgM to all three house dust mite extracts and no scabies patient had antibodies exclusively to scabies mites. Thus, the key to a diagnostic test that possesses both specificity and sensitivity likely lies in identifying a limited and defined set (used as a cocktail) of scabies proteins (or protein fragments) that do not carry epitopes that cross-react with those on house dust mite proteins and that bind IgM from the serum of scabies patients. To accomplish this goal, a detailed and comprehensive proteomic and genomic analysis of *S*. *scabiei* is necessary.
